# Harnessing Fiber Diameter-Dependent Effects of Myoblasts Toward Biomimetic Scaffold-Based Skeletal Muscle Regeneration

**DOI:** 10.3389/fbioe.2020.00203

**Published:** 2020-03-24

**Authors:** Naagarajan Narayanan, Chunhui Jiang, Chao Wang, Gözde Uzunalli, Nicole Whittern, Da Chen, Owen G. Jones, Shihuan Kuang, Meng Deng

**Affiliations:** ^1^Department of Agricultural and Biological Engineering, Purdue University, West Lafayette, IN, United States; ^2^Bindley Bioscience Center, Purdue University, West Lafayette, IN, United States; ^3^Department of Animal Sciences, Purdue University, West Lafayette, IN, United States; ^4^Department of Food Sciences, Purdue University, West Lafayette, IN, United States; ^5^Weldon School of Biomedical Engineering, Purdue University, West Lafayette, IN, United States; ^6^Department of Materials Engineering, Purdue University, West Lafayette, IN, United States

**Keywords:** polyesters, myoblasts, topography, electrospun fibers, skeletal muscle

## Abstract

Regeneration of skeletal muscles is limited in cases of volumetric muscle loss and muscle degenerative diseases. Therefore, there is a critical need for developing strategies that provide cellular and structural support for skeletal muscle regeneration. In the present work, a bioengineered cell niche composed of mechanically competent aligned polyester fiber scaffolds is developed to mimic the oriented muscle fiber microenvironment by electrospinning poly(lactide-*co*-glycolide) (PLGA) using a custom-designed rotating collector with interspaced parallel blades. Aligned fiber scaffolds with fiber diameters ranging from 335 ± 154 nm to 3013 ± 531 nm are characterized for their bioactivities in supporting growth and differentiation of myoblasts. During *in vitro* culture, polymeric scaffolds with larger fiber diameter support enhanced alignment, growth, and differentiation of myoblasts associated with phosphorylation of p38 MAPK and upregulated expression of myogenin and myosin heavy chain. *In vivo* studies using a dystrophin-deficient mdx mouse model show that optimized fiber scaffolds seeded with primary myoblasts result in formation of dystrophin-positive myofibers network in tibialis anterior muscles. Collectively, these experiments provide critical insights on harnessing interactions between muscle cells and engineered fiber matrices to develop effective biomaterials for accelerated muscle regeneration.

## Introduction

Skeletal muscles are composed of oriented muscle fibers that are formed by the fusion of myoblasts. This orientation in fiber alignment enables an anisotropic organization of extracellular matrix (ECM) for functional contraction, which leads to bodily movement. In response to minor injuries, skeletal muscles exhibit a remarkable regenerative capacity empowered by a unique stem cell population, satellite cells. Satellite cells are localized along the surface of muscle fibers under the basal lamina, offering a niche microenvironment that regulates the cell functions (Kuang et al., 2008). Following muscle injuries, satellite cells get activated, enter the cell cycle, migrate to the injury site, and proliferate. A portion of the proliferating cells (called myoblasts) then withdraw from the cell cycle and differentiate to form mature muscles, whereas another portion of the myoblasts self-renew and return to quiescence (Kuang et al., 2007). Unfortunately this natural repair mechanism by satellite cells is interrupted in conditions such as muscle degenerative diseases or volumetric muscle loss (Turner and Badylak, 2012). Consequently, cell-based therapies involving the injection of myogenic cells have been investigated to treat skeletal muscle injuries and diseases. However, complications due to low survival and poor long-term engraftment of the transplanted cells hinder the use of cell-based therapies (Qu et al., 1998; Joe et al., 2010; Negroni et al., 2011; Briggs and Morgan, 2013). There is a critical need for developing strategies that can provide cellular and structural support by mimicking the skeletal muscle cell niche to aid in the regeneration of functional skeletal muscles.

**Figure 1:**
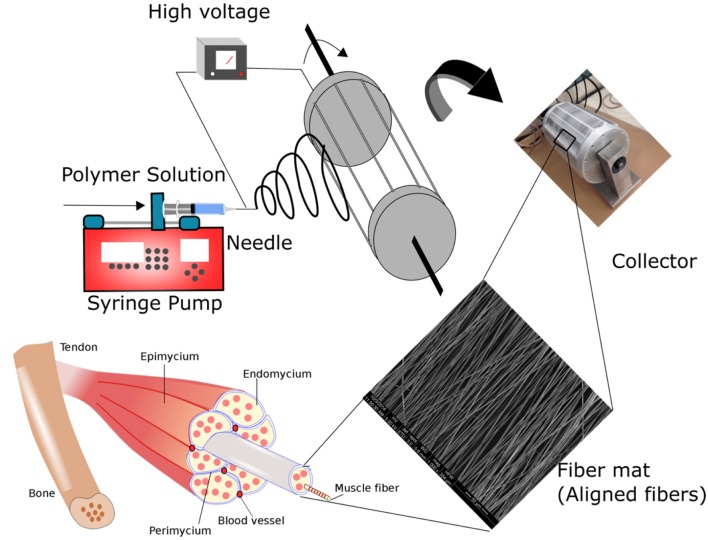
Schematic illustrating the fabrication of polymer fibers via electrospinning. In the process of electrospinning, fibers are created by providing electric potential to the polymer solution placed in the syringe pump. Polymer jets are ejected from the tip of the needle when an electric potential greater that the surface tension of the polymer solution is applied. Ultra-thin polymer fiber structures are formed due to bending and stretching instabilities of the polymer jet. By varying the process parameters, fibers with varying morphology and structure can be fine-tuned. Skeletal muscle is characterized by oriented myofibers bundled together to form the tissue architecture. In the study, polymer fibers mimicking the native skeletal muscle microenvironment were fabricated by collecting the fibers on a custom-designed rotating drum collector. The novel drum collector has interdigitated blades to provide an enhanced local electric field to improve the alignment of the polymer fibers.

Tissue engineering offers a transdisciplinary strategy for skeletal muscle regeneration by using scaffolds, cells, and growth factors alone or in combination (Deng et al., 2012; Narayanan et al., 2016). An ideal scaffold for skeletal muscle tissue should mimic the anisotropic organization of muscle ECM and present a biocompatible and cell-instructive microenvironment to guide cell behavior and augment muscle regeneration. Specifically, aligned polymer fiber structures have attracted significant research interests as muscle-mimicking scaffolds due to their resemblance to skeletal muscle cell niche. For example, aligned fibers have been demonstrated to provide favorable contact guidance for myoblasts, and facilitated cell-material interactions and myoblast alignment when compared to random fibers (Yoshizato et al., 1981; Aviss et al., 2010; Zhang et al., 2012). These studies have provided important insights into the effect of fiber alignment and topography on myoblast behavior. Besides fiber alignment, curvature presented by the polymer fibers also play a critical role in modulating cellular behavior (Badami et al., 2006; Christopherson et al., 2009; Cardwell et al., 2014; Hodgkinson et al., 2014; Bean and Tuan, 2015). Previous studies have employed fiber diameters of ~300 nm (San Choi et al., 2008), ~600–900 nm (Aviss et al., 2010), ~1,000–3,000 nm (McKeon-Fischer and Freeman, 2011), as well as ~10 μm (Riboldi et al., 2005) for potential skeletal muscle tissue engineering applications. The way in which myoblasts sense and respond to the fiber diameter differences, and translate to skeletal muscle regeneration remains to be investigated. Therefore, understanding the topographical cues presented by the polymer fibers could provide critical insights into tailoring aligned fiber structures for development of cell-instructive biomaterials to promote myoblast responses and skeletal muscle regeneration. Inspired by the native architecture of skeletal muscle, we aim to develop a bioengineered cell niche by mimicking the oriented muscle fiber structures to facilitate skeletal muscle regeneration. In specific, we have designed an electrospinning setup (Figure 1) to produce aligned electrospun polymer fibers with fiber diameters varying from 335 ± 154 nm to 3013 ± 531 nm. Electrospinning is a versatile technology platform for fabricating ultra-thin polymer fibers with varying architecture and topography by modulating its process parameters (Deng et al., 2011, 2012). A drum roll collector has been modified with parallel blades that are equally spaced to provide a local uniform electric field to enhance the alignment of the deposited fibers. A synthetic aliphatic polyester, poly(lactide-*co*-glycolide) (PLGA, 85:15 lactide to glycolide ratio), was specifically selected to produce fiber scaffolds due to its biodegradability, biocompatibility and desirable mechanical properties for skeletal muscle engineering (Deng et al., 2012; Narayanan et al., 2016). The fabricated fibers were tested *in vitro* to optimize the fiber curvature in promoting cell-material interactions involving adhesion, proliferation, and differentiation. *In vivo* study using an *mdx* mouse model were conducted to evaluate the regeneration efficacy of the optimized PLGA fiber scaffolds.

## Materials and Methods

### Materials

Poly(lactide-*co*-glycolide) (PLGA) 85:15 (94 kDa) was purchased from Lakeshore Biomaterials. Tetrahydrofuran (THF), N, N-Dimethylformamide (DMF), Triton X-100 and 4% paraformaldehyde was obtained from Fischer Scientific. High Glucose DMEM with L-Glutamine and sodium pyruvate, and Penicillin/Streptomycin was purchased from Gibco, Life Technologies. Fetal bovine serum (FBS) and horse serum were acquired from Atlanta Biologicals. FAK-100 actin cytoskeleton kit (EMD Millipore) was employed for actin staining. Fluorescent mounting medium was obtained from Dako, Agilent Technologies and Pico green assay kit was purchased from Life Technologies.

### Electrospinning

PLGA [20-40% (w/v)] solution in THF: DMF (3:1) were loaded into a 10-mL syringe fitted with an 18G blunt needle. The needle was connected to a power supply of 20 kV. The polymer solution was fed at a rate of 2 mL/h. A flat plate collector was used for the fabrication of random fibers. A custom-designed drum roller with interdigitated blades was employed for the fabrication of aligned fibers (Figure 1). The digitated blades were equally spaced to provide the required local electric field for the alignment of the fibers within the blades (Figure 1). The collector was placed at a distance of 20 cm to maintain a potential of 1 kV/cm. Aligned fibers were collected at a rotation speed of 1,000 rpm for 5 h. The entire electrospinning set up was placed inside a cabinet with an outlet for the solvent evaporation. Random PLGA fibers were fabricated employing a flat plate collector using 30% PLGA. Process parameters for the fabrication was set at 2 mL/h flow rate, 1 kV/cm electric potential and time of 5 h.

### SEM Characterization of Aligned Fibers

Polymer solutions with different concentrations were electrospun for 30 min, the fibers were mounted directly onto the SEM stubs using carbon tape. The samples were coated with platinum using a Cressington turbo-pumped sputter coater for 30 s and analyzed using FEI NOVA nanoSEM FESEM. The surface morphology of the fiber samples was imaged at various magnifications using 5 kV accelerating voltage. The fiber diameters were measured, and their alignment was characterized based on the angle of individual fibers with respect to the average alignment (baseline of 0° is along the alined axis of the electrospun fibers) of the fiber samples using Image J software (*n* = 90 fibers; *n* = 3 images per sample).

### Cell Culture

C2C12 murine myoblast cells were cultured using media consisting of high glucose DMEM supplemented with 10% FBS and 1% penicillin/streptomycin. Routinely cells were seeded on a petri dish and grown in a humidified incubator maintained at 37°C, 5% CO_2_. For the scaffold-based studies, the fiber scaffolds (cut into equal sizes) were immobilized using Scaffadex 48-well plate inserts as previously reported (Aviss et al., 2010). Cells were seeded at 1 × 10^4^ cells per scaffold for cell adhesion and proliferation.

### Cell Adhesion

The morphology and alignment of the cells was characterized by staining the actin cytoskeleton. Cells harvested at 2 h and 24 h were fixed in 4% paraformaldehyde (PFA) before being permeabilized with 1% Triton X-100. The fixed cells were blocked using 1% bovine serum albumin (BSA) followed by staining for F-actin (TRITC-conjugated phalloidin primary F-actin antibody) and nuclei (DAPI). The scaffolds with stained cells were mounted on a glass slide with anti-fade mounting media and imaged using EVOS FL imaging system. The cell elongation ratio, cell spreading area, and cell alignment (*n* ≥ 120 cells across *n* ≥ 4 images per sample) were characterized as previously reported using Image J software (Wang et al., 2015). At predetermined time points of day 1, day 3, and day 7 post cell seeding, the cells seeded on scaffolds were fixed using 4% PFA. The scaffolds were dehydrated using a series of ethanol treatments (50, 70, 80, 90, and 100%) with an incubation time 10 min for each treatment. The dehydrated scaffolds were mounted on a SEM stub using a carbon tape. The samples were coated with platinum using a Cressington turbo-pumped sputter coater for 30 s. The coated samples were imaged using FEI NOVA nanoSEM FESEM.

### Cell Proliferation

Cell proliferation was quantified by using Pico Green assay as instructed by the manufacturer (Life Technology, USA). The assay involves the quantification of double-stranded nucleic acids using an ultra-sensitive fluorescent probe. The intracellular content was extracted from each scaffold sample using 1% Triton X-100 in 1 × PBS. To completely rupture the cell membrane and extract the nucleic acid content, samples were freeze-thawed three times. The obtained solution was used for the quantification of the double stranded nucleic acid content (Deng et al., 2010b).

### Cell Differentiation

For differentiation studies, cells were seeded at 1 × 10^5^ cells per scaffold and incubated in growth media for 24 h. After which the media was changed to differentiation media consisting of high glucose DMEM supplemented with 2% horse serum and 1% penicillin/streptomycin.

After 7 days of cell differentiation, the samples were harvested and fixed in 4% PFA. The fixed samples were permeabilized using 1% Triton-X followed by the 1% BSA blocking. The primary antibody was added at a dilution of 1:20 for the myosin heavy chain (MHC) (MF 20, Developmental Studies Hybridoma Bank, University of Iowa). The samples were incubated overnight at 4°C followed by washing with 1 × PBS three times before the addition of the FITC labeled secondary antibody. The samples were washed three times with 1 × PBS to remove excess secondary antibody. The stained samples were mounted on glass slides along with fluorescent mounting media. The samples were imaged using an Olympus FV10i confocal microscope at different magnifications.

### Western Blot

Samples (*n* = 3) were combined to extract enough total protein using RIPA buffer. The protein concentration was quantified with Pierce BCA protein reagent (Pierce Biotechnology, Rockford, IL, USA). SDS-polyacrylamide gel electrophoresis was performed to the separate proteins, after which they were transferred to polyvinylidene fluoride (PVDF) membranes (Millipore Corp., Billerica, MA, USA). Membranes were blocked with 5% BSA in Tris-buffered saline—Tween 20 (TBST) buffer. Antibodies used were the following: Phospho-p38 MAPK (Thr180/Tyr182) (Cell signaling technology; 1:1000 dilution), Phospho-FAK (Tyr397) (Cell signaling technology; 1:1000 dilution) and MyoG F5D (Developmental Studies Hybridoma Bank, University of Iowa; 1:1000 dilution). The secondary antibody (anti-rabbit IgG for Phospho-p38 MAPK and Phospho-FAK; anti-mouse IgG for MyoG) conjugated with horseradish peroxidase was applied (1:5000). ChemiDoc™ Touch Imaging System (Bio-Rad) was utilized to detect the protein bands.

### Primary Myoblast Isolation and Culture

Primary myoblasts from mice samples were isolated as described in a previous study (Shan et al., 2014). Briefly, skeletal muscle in the hind limb was collected and minced. The minced muscle tissue was digested with type I collagenase and dispase B digestion mixture. Ham's F10 media supplemented with 20% FBS was used to stop the digestion. The digested muscle tissue was centrifuged at 450 g for 5 min. The obtained primary myoblasts were maintained in collagen-coated dishes with a growth medium consisting of Ham's F10 (20% FBS, 4 ng/mL basic fibroblast growth factor, and 1% penicillin/streptomycin).

### *In vivo* Implantation Study

*Mdx* mice were purchased from the Jackson Laboratory (stock #001801). Mice were housed in the animal facility with free access to water and standard rodent chow. All procedures involving the use of animals were performed in accordance with the guidelines presented by Purdue University's Animal Care and Use Committee. Without special mentioning, we used 2-month-old male mice for experiments. Cardiotoxin (CTX) injection (10 μM) was administered in the tibialis anterior (TA) muscle of *mdx* mice 24 h prior scaffold implantation. 2 × 10^5^ primary myoblasts were seeded on Matrix 3 fibers and cultured overnight in growth media. Scaffolds (2 mm in diameter) were implanted at the site of CTX administration. Myoblasts cells (2 × 10^5^ cells in 500 μL primary myoblast growth medium) were injected at the site of CTX administration and were used as controls. The TA muscle, for both control and scaffold group, was harvested by cryo-fixing the samples in OCT 3 weeks after implantation. Cryosections of the TA muscles were stained for dystrophin (ab15277, Abcam; 1:1000 dilution) for further analysis. The stained cross-sections (230 × 230 μm) were imaged in Nikon A1R MP confocal microscopy and quantified to evaluate dystrophin positive myofibers per cross section image obtained (*n* = 3 per group).

### Statistical Analysis

Quantitative data were reported as mean ± standard deviation (SD). Statistical analysis was performed using a one-way analysis of variance (one-way ANOVA). Comparison between means was determined using the Tukey *post-hoc* test with a minimum confidence level of *p* < 0.05 for statistical significance. Student *t*-test was used to compare experimental groups for the *in vivo* dystrophin staining analysis with confidence interval for *p* < 0.05 for statistical significance.

## Results

### Fabrication and Characterization of Aligned Fibers

**Figure 2:**
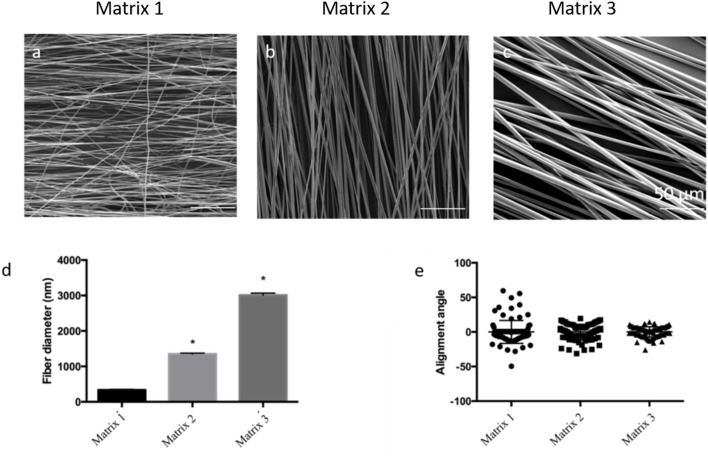
SEM images for the morphological analysis of aligned PLGA fibers with different polymer concentrations **(a)** 20% PLGA (Matrix 1); **(b)** 30% PLGA (Matrix 2); **(c)** 40% PLGA (Matrix 3); Scale bar 50 μm. **(d)** Bar graphs of the relationship between PLGA fiber diameter and polymer concentration (*n* = 90 fibers; *n* = 3 images per scaffold) showing that an increase in PLGA concentration from 20 to 40% resulted in an increase of fiber diameter ranging from 335 ± 154 nm to 3013 ± 531 nm. *p < 0.05 indicates significant difference as compared to Matrix 1; **(e)** Categorical scatter plot showing mean alignment angle with the spread for PLGA fibers indicating high alignment of fibers using three different polymer concentrations. PLGA concentrations below 20% resulted in bead formation, whereas PLGA concentrations above 40% were too viscous to be electrospun.

In the skeletal muscles, ECM protein molecules have a well-defined structural organization creating tightly packed and highly oriented fibers to facilitate specific skeletal muscle functions. Previous investigations have suggested that topography plays a critical role in modulating functional and structural properties of the cells (Yoshizato et al., 1981; Aviss et al., 2010). Accordingly, we have designed and fabricated aligned PLGA fibers to mimic the natural topography of skeletal muscle ECM. Aligned fibers were produced by employing an electrospinning setup with a custom-designed collector (Figures S1a,b) (Zhang et al., 2012). This custom design integrates equally spaced metal blades present on the surface of the collector and increases the local electric field between the blades, thereby improving the alignment of the fiber deposited between them (Figure S1b). By varying the PLGA concentration (w/v), we have successfully fabricated three types of aligned fiber matrices with different fiber diameters, namely Matrix 1 (20%PLGA), Matrix 2 (30%PLGA), and Matrix 3 (40%PLGA). The fabricated fibers, characterized by scanning electron microscopy (SEM), were found to be highly uniform with smooth surfaces (Figures 2a–c, Figures S1c–e). The average fiber diameter was 335 ± 154 nm, 1352 ± 225 nm and 3013 ± 531 nm for Matrix 1, Matrix 2, and Matrix 3, respectively (Figure 2d). The average alignment angle was 16.6°, 11.1°, and 7.4° for Matrix 1, Matrix 2, and Matrix 3, respectively (Figure 2e).

Surface topography of a single fiber for different fiber matrices was analyzed by AFM AC tapping mode. Single fibers revealed by AFM supported the SEM imaging for the morphology and fiber diameters of electrospun fiber matrices (Figures S2a–c). Surface stiffness is an important physical parameter to consider when designing scaffolds for cell-biomaterial interactions and skeletal muscle engineering. AFM nanoindentation studies were performed to characterize surface stiffness of fiber matrices with varying fiber diameters. The elastic modulus was calculated based on Oliver Pharr model and tabulated as shown in Figures S2d,e. The elastic modulus values were found to be 383 ± 53 MPa, 317 ± 67 MPa, and 371 ± 38 MPa for Matrix 1, Matrix 2, and Matrix 3, respectively. Statistical analysis demonstrated no significant changes in stiffness of PLGA fibers with diameters varying from 335 ± 154 nm to 3013 ± 531 nm.

### Effect of Fiber Diameter on Myoblast Adhesion and Proliferation

**Figure 3:**
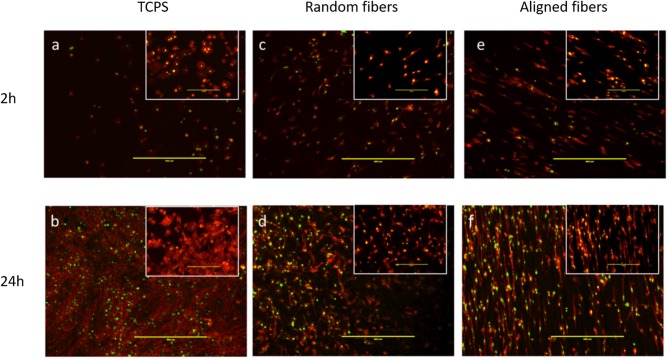
Representative fluorescence images via immunostaining of C2C12 myoblasts showing cell adhesion on **(a,b)** TCPS, **(c,d)** random electrospun PLGA fibers and **(e,f)** aligned electrospun PLGA fibers (Matrix 2). Top panel illustrates images taken 2 h post cell seeding whereas bottom panel illustrates images taken 24 h post cell seeding. Inserts illustrate higher magnification images. Myoblasts seeded on aligned PLGA fibers exhibited oriented arrangement along the direction of fiber alignment resulting from cell contact guidance as compared to those seeded on TCPS and random PLGA electrospun fibers (Red, actin cytoskeleton; Green, nuclei; Scale bar, 400 μm).

**Figure 4:**
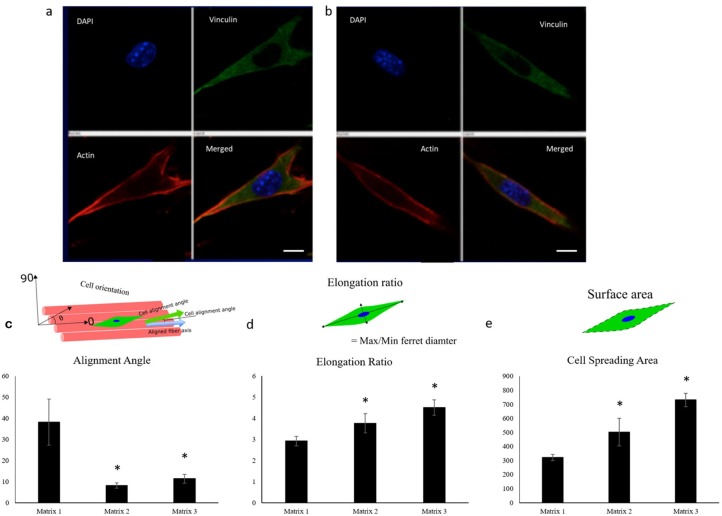
Representative confocal images of C2C12 cells showing the cell elongation 2 h post seeding on **(a)** Matrix 1 and **(b)** Matrix 3 demonstrating the ability of myoblasts to sense differences in fiber diameters (Red, actin cytoskeleton; Blue, nuclei; Green, vinculin). Scale bar 10 μm. Quantification of **(c)** alignment angle, **(d)** elongation ratio, and **(e)** cell spreading area of seeded myoblasts 2 h post seeding showing enhanced myoblast alignment, elongation ratio, and cell spreading area on the Matrix 2 and Matrix 3 as compared to the Matrix 1. *n* ≥ 4 images for each sample. *p < 0.05 as compared to Matrix 1.

C2C12 cells, an established murine myoblast cell line, were seeded and cultured on fiber matrices to characterize effects of fiber diameter on myoblast adhesion and growth. As evidenced from fluorescence microscopy images, alignment of the PLGA fibers influenced myoblast polarity and improved cell orientation compared to random fibers and tissue culture polystyrene (TCPS) controls at 2 and 24 h post-seeding (Figure 3). Interestingly, aligned fibers resulted in elevated phosphorylation of focal adhesion kinase (FAK), which is a cytoplasmic non-receptor tyrosine kinase important for transmitting extracellular cues to intracellular targets (Figure S3). This find is in line with literature reports on role of FAK in topography effects on cell-biomaterial interactions (Kuang et al., 2019). Further analysis was performed to characterize the effect of fiber diameter on cell alignment, elongation ratio and cell spreading area using confocal images of C2C12 cells immunostained for actin, vinculin, and nuclei. C2C12 cells on Matrix 3 exhibited a classic spindle (bipolar) morphology as compared to those on Matrix 1 (Figures 4a,b).

**Figure 5:**
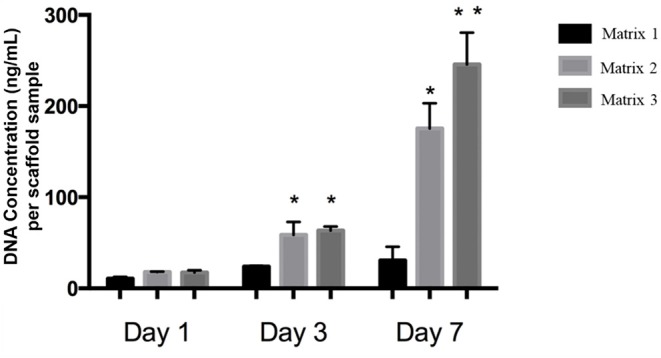
DNA concentrations of C2C12 cells on different fiber matrices during cell culture illustrating increased cell proliferation on Matrix 2 and Matrix 3 as compared to Matrix 1. Matrix 3 supported highest cell numbers after 7 days of culture. *n* = 3 for each group. *p < 0.05 as compared to Matrix 1. **p < 0.05 as compared to both Matrix 1 and Matrix 2.

Quantitatively, Matrix 2 and Matrix 3 supported greater cell alignment as compared to Matrix 1 as evidenced from the alignment angle analysis after 2 h of cell seeding (Figure 4c). Furthermore, myoblasts seeded on larger fibers exhibited a significant increase in elongation ratio (1.3-fold for Matrix 2 and 1.5-fold for Matrix 3) as well as cell spreading area (1.6-fold for Matrix 2 and 2.2-fold for Matrix 3) as compared to those seeded on Matrix 1 (Figures 4d,e), indicating the curvature sensing ability of cells to fiber matrices. The responses of C2C12 cells in contact with polymer fibers were further examined by SEM to monitor the cell morphology after initial cell attachment and spreading on surfaces. After 24 h, cells adhered on the fiber surfaces in all three groups, thereby indicating that the aligned synthetic fibers supported cell attachment (Figures S4a–c). As the cells continued to proliferate on fiber matrices, cell coverage increased. At day 3, increased numbers of cells were observed on the surfaces of Matrix 2 and Matrix 3 than on the surface of Matrix 1 (Figures S4d–f). By day 7, the surfaces of the Matrix 2 and Matrix 3 were uniformly covered by aligned myoblasts whereas open areas on the surface of Matrix 1 were still visible (Figures S4g–i). Proliferation of C2C12 cells on the electrospun fiber matrices was quantified by measuring the DNA content of the cells using PicoGreen dsDNA assay. Consistent with SEM micrographs, both Matrix 2 and Matrix 3 supported progressive myoblast growth with increasing culture time (Figure 5). Furthermore, larger-diameter fiber matrices showed a significant increase in cell numbers (3.4-fold for Matrix 2 and 3.6-fold for Matrix 3 after 3 days; and 5.7-fold for Matrix 2 and 8.0-fold for Matrix 3 after 7 days) than Matrix 1, suggesting that increase in fiber diameter improved cell growth.

### Myoblast Differentiation on Aligned Fibers

**Figure 6:**
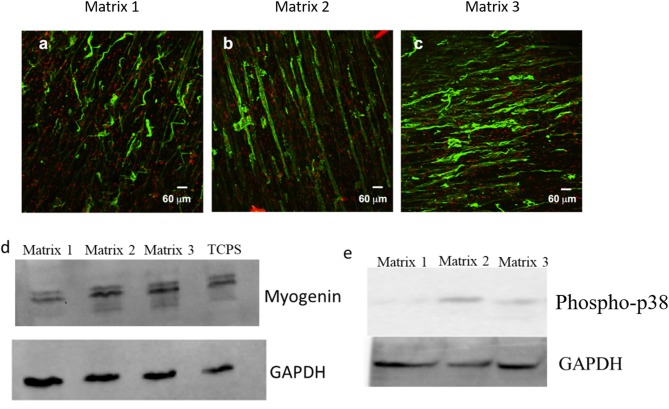
Representative confocal images for myotubes immunostained for MHC (myogenic maturity marker) after 7 days of differentiation on **(a)** Matrix 1, **(b)** Matrix 2, and **(c)** Matrix 3 showing extensive aligned myotubes on Matrix 2 and Matrix 3 as compared to distorted myotubes on Matrix 1. Western blot analysis for **(d)** myogenin (mid-stage myogenic marker), and **(e)** phospho-p38 showing an elevated protein expression of C2C12 cells on larger fiber matrices.

*In vitro* differentiation studies of C2C12 cells on aligned fibers were performed to assess the effects of fiber diameter on myoblast differentiation. In specific, differentiation of myoblasts into myotubes was characterized by immunofluorescent staining for the myotube maturation marker, myosin heavy chain (MHC). As shown in Figures 6a–c, robust staining for MHC in aligned myotubes was detected for Matrix 2 and Matrix 3, whereas distorted MHC staining for myotubes was observed on Matrix 1 after 7 days of culture. Additionally, anisotropic organization of myotubes similar to native skeletal muscle architecture was observed on the Matrix 2 and Matrix 3. Furthermore, western blot analysis demonstrated that myogenin expression levels were also significantly elevated in myotubes formed on Matrix 2 and Matrix 3 as compared to Matrix 1 (Figure 6d). In addition, we examined levels of p38, a mitogen-activated protein kinases (MAPK) family member that is activated via external cues such as fiber diameter/curvature, in myoblasts seeded on fiber matrices (Jaiswal and Brown, 2012). The results showed that phospho-p38 expression levels in myoblasts on Matrix 2 and Matrix 3 were greater as compared to those on Matrix 1 (Figure 6e). This revelation suggested that large fibers presented favorable topographical contact guidance via activation of p38 MAPK thereby facilitating in differentiation.

### *In vivo* Evaluation

**Figure 7:**
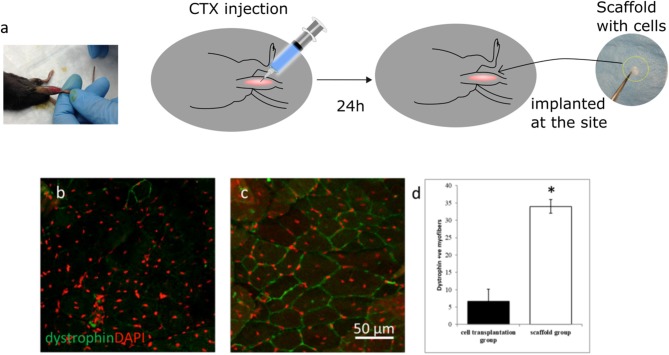
**(a)** Schematics illustrating the scaffold implantation procedure in the TA muscle of *mdx* mice. Representative confocal micrographs of TA muscle sections after 21 days of **(b)** myoblast injection and **(c)** implantation of scaffolds seeded with myoblasts. **(d)** Quantification of dystrophin positive myofibers shows a significant increase in positive myofibers for the scaffold group when compared to the myoblast transplantation group. Fiber scaffolds seeded with primary myoblasts were able to generate a significant number of dystrophin positive myofibers indicating enhanced myogenic potential whereas presence of dystrophin positive myofibers was scarce for the myoblast transplantation group. *n* = 3 for each group. *p < 0.05 as compared to cell transplantation group.

An *mdx* mouse model was used to evaluate the *in vivo* regeneration efficacy of aligned fiber scaffolds. These mice lack native dystrophin, a structure protein in the cell membrane, making dystrophin an appropriate biomarker for *in situ* regenerative efficacy of transplanted cells through immunostaining of dystrophin-positive myofibers at the injury site. Matrix 3 was selected for the *in vivo* study due to enhanced myoblast proliferation and differentiation *in vitro*. As shown in Figure 7a, acute skeletal muscle injury was first induced in the tibialis anterior (TA) muscles of *mdx* mice with cardiotoxin (CTX) treatment followed by implantation of scaffolds seeded with primary myoblasts. To determine the regeneration improvements by scaffold-mediated cell grafting, myoblast transplantation alone was used as controls. After 21 days of transplantation, presence of dystrophin-positive myofibers was scarce in the myoblast transplantation group (Figure 7b, Figure S5a), however robust networks of dystrophin-positive myofibers were observed in the scaffold implantation group (Figure 7c, Figure S5b). Quantification analysis showed that there was a significant increase in dystrophin-positive myofibers at the injury site for the scaffold implantation group compared to the myoblast transplantation group (Figure 7d). These results suggest that the fiber scaffolds provided the required structural and cellular support for the seeded myoblasts, leading to the formation of dystrophin-positive myofiber networks.

## Discussion

Living cells dynamically interact with their surrounding microenvironment which subsequently modulates cell functions (Rosso et al., 2004). Specifically, oriented muscle fibers and their ECM components form a unique cellular niche in skeletal muscle tissue (Kuang et al., 2008). Successful skeletal muscle regeneration necessitates the design and development of biomimetic aligned fiber scaffolds that serve as temporary substrates to support enhanced cellular activities and matrix synthesis. In a scaffold-based regeneration process, the scaffold is designed to initially provide mechanical support and gradually the scaffold is replaced by the newly synthesized ECM at the injury or defect site. PLGA polymers constitute an attractive class of biodegradable polymers to develop 3D scaffolds due to their FDA approval for a number of clinical applications (Deng et al., 2012; Narayanan et al., 2016). Scaffold degradation and mechanical properties can be tailored by altering the lactide:glycolide ratio. Among various ratios of copolymers, PLGA 85:15 was selected based on mechanical competence and relatively slow degradation in the range of 5–6 months, which is advantageous to sustain muscle function during regeneration (Middleton and Tipton, 2000).

A custom-made electrospinning process was utilized to produce aligned PLGA fibers in nanometer as well as micrometer ranges by integrating the advantages of both the mechanical and electrical forces (Figure S1). Our new rotating collector incorporated with the modular design of metal blades (e.g., variable gap sizes) provides a local electric field within the blades to facilitate the alignment of the deposited fibers, thereby enabling the precise fabrication of anisotropic scaffolds that match the size of an injured muscle. The fiber diameter and alignment can be effectively controlled by changing various electrospinning and process parameters including polymer solution concentration, rotating speed, and electrical potential. For example, a series of polymer concentrations were used for electrospinning PLGA (20–40% w/v) to produce fiber diameters from a few 100 nanometers to a few microns. The increase of polymer concentration resulted in an increase in the fiber diameter and larger fiber size distribution. The ability to generate fibers with varying diameters allowed us to study the fiber-diameter-dependent cellular responses of myoblasts. It has been well-documented that scaffold mechanical properties also influence myoblast responses (Levy-Mishali et al., 2009). It was confirmed by AFM nanoindentation that all fiber matrices showed similar elastic moduli, which were within the range of reported values for PLGA films and electrospun PLGA fibers (Moffat et al., 2008; Deng et al., 2010a). For example, the elastic modulus for aligned PLGA scaffolds with average fiber diameter of 615 nm was reported to be ~341 MPa. The as-fabricated PLGA fiber matrices composed of varying fiber diameters (from 300 nm to 3000 nm) were systemically evaluated for optimal cell-material interactions and *in situ* skeletal muscle regeneration. To our best knowledge, there is not a reported comparative study on optimizing the effects of this PLGA fiber diameter range on muscle cell responses. Larger diameter fibers were not included since cells on large diameter substrates (>10 μm) resemble those on planar surfaces (Hwang et al., 2009).

Myoblast adhesion involving specific alignment of myoblasts is one of the key elements in initial cell-material interactions affecting regeneration of functional skeletal muscles (Shimizu et al., 2009; Wang et al., 2015). Therefore, anisotropically oriented substrates with contact guidance capacity is desirable for myoblast adhesion and alignment (Charest et al., 2007; Duan and Gallagher, 2009; Guex et al., 2012; Nikkhah et al., 2012; Wang et al., 2015). The electrospun fiber matrices produced in this study were able to support cytoskeleton reorganization to orient the cells according to the direction of the fiber alignment (Figures 3, 4). Aligned polymer fibers induced a significant increase in pFAK expression levels of seeded myoblasts compared to random polymer fibers (Figure S3). Phosphorylated FAK has been documented to induce further signaling cascades (Kuang et al., 2019). For example, phosphorylation of FAK activates tyrosine kinase Src, which further triggers the activation of RhoA and ROCK complexes to mediate cytoskeleton remodeling (McMurray et al., 2015). These results are in line with previous reports that indicated focal adhesion plays an important role in cell orientation on patterned surfaces for fibroblasts and osteoblasts (Ventre and Netti, 2016). Furthermore, topographical cues presented by differential fiber diameters influenced myoblast cell adhesion and growth (Figures 4, 5). Specifically, myoblasts cultured on Matrix 2 and Matrix 3 exhibited classical bipolar structures as well as greater cell alignment, elongation ratio and cell spreading area as compared to those on Matrix 1. This finding demonstrated that the topography of the larger fibers supported favorable cell-matrix interactions to promote cell adhesion events, which indicated the ability of C2C12 cells to sense and respond to the degree of curvature of fibers (Vogel and Sheetz, 2006). The cell density on larger-diameter fiber matrices was greater than the cell density on Matrix 1 after 7 days of culture. Badami et al. reported a similar finding using electrospun PLA fibers that cell density increased with fiber diameter, which was attributed to the favorable properties of larger-diameter fibers (2,100 nm) in permitting cell infiltration and tissue formation as compared to smaller-diameter fibers (140 nm) (Badami et al., 2006). However, as the pore size increases with increased fiber diameter and if the pores get too large (>20 μm), cells tend to grow along single fibers instead of in a 3D configuration, which could hinder cell growth and ECM production. Both enhanced alignment and proliferation of C2C12 cells on Matrix 3 observed in the current study suggest that Matrix 3 provide appropriate scaffold fiber diameter and architecture to achieve the balance between cell alignment and cell proliferation (Lowery et al., 2010; Bean and Tuan, 2015).

Following cell adhesion, myogenesis is largely dependent on cell differentiation and myoblast fusion that leads to the formation of multinucleated myotubes. In particular, generation of mature myotubes is critical for functional regeneration of skeletal muscles (Chal and Olivier, 2012). In this study, we showed that Matrix 2 and Matrix 3 enhanced myoblast differentiation and fusion (Figure 6). Notably, myoblasts seeded on those fiber matrices showed an elevated expression of phosphorylated p38, an important regulator of myoblast differentiation through the p38 mitogen-activated protein kinases (MAPK) signaling pathway (Cuenda and Rousseau, 2007). p38 MAPK/Erk activation through phosphorylation is related to the integrin-mediated downstream pathway that is triggered via external stimuli (Roux and Blenis, 2004). It has been shown that p38 in myoblasts phosphorylates myocyte enhancement factor 2 (MEF-2), an important transcription factor which binds to myogenic regulatory factor (MRF) to trigger myogenic genes (Zhao et al., 1999). Furthermore, we showed that myotubes generated on Matrix 2 and Matrix 3 had robust staining for myotube maturation marker MHC, which is an essential motor protein involved in muscle contraction and relaxation (Wells et al., 1996). We have also shown an increase in differentiation marker myogenin of myoblasts cultured on Matrix 2 and Matrix 3 compared to Matrix 1. These *in vitro* results collectively suggested that larger-diameter fibers presented favorable topographical contact guidance to facilitate myoblast differentiation and mature myotube formation.

Myoblast transplantation therapy has been proposed as a potential treatment for Duchene muscular dystrophy and other muscle related disorders (Gussoni et al., 1992; Skuk et al., 2006; Sienkiewicz et al., 2015). To date, the isolated primary myoblasts are injected directly into the muscle tissue; however, the transplanted cells suffer from limited cell survivability. More importantly, injection of myoblasts and transplantation of disoriented muscle flaps may not integrate well within the host tissue, thereby resulting in poor biomechanical functions (Kuang and Rudnicki, 2008; Corona et al., 2015). As a first step of efficacy evaluation toward translation of our scaffold technology, *mdx* mice were used as a model. The selection of aligned scaffolds composed of larger fibers was based on comparative *in vitro* studies by optimizing the alignment and fiber diameter effects on myoblast responses. To assess the *in vivo* myogenic potential of optimized fiber scaffolds, we transplanted primary myoblasts either with or without the scaffolds in the TA muscle of *mdx* mice. Here, we showed that the Matrix 3, when seeded with primary myoblasts, was well-tolerated and induced formation of a network of dystrophin-positive myofibers following 21 days of transplantation. In contrast, transplantation of myoblasts without scaffolds failed to generate a significant population of dystrophin-positive myofibers, due to poor cell survivability and integration, consistent with previous studies (Briggs and Morgan, 2013). To overcome the limitations of current cell-based therapies, efforts have been made involving implantation of high-dose cells or multiple cell delivery (Kim et al., 2016). However, higher cell density within the implanted area (e.g., 30 × 10^6^ cells per ml) often result in higher necrosis due to limited diffusion of oxygen and nutrients supplied by the host. The encouraging *in vivo* evidence presented in this study for our scaffold-based transplantation approach attests to the promise in developing bioengineered transplantable systems that foster the survival and function of myogenic cells to promote myogenesis for *in situ* skeletal muscle regeneration. In particular, combining a versatile electrospinning process with FDA-approved PLGA will facilitate the clinical translation of biodegradable and biomimetic scaffolds for skeletal muscle repair and regeneration applications. Current ongoing studies focus on efficacy evaluation of this fiber scaffold platform for skeletal muscle regeneration using a volumetric muscle loss model.

## Conclusion

In the present work, biomimetic structures composed of aligned electrospun polyester fibers were fabricated and characterized to optimize fiber diameter effects on myoblasts for skeletal muscle regeneration. We demonstrated that alignment of the scaffold fibers promoted FAK phosphorylation and reorientation of the actin cytoskeleton leading to elongated cell morphology. Interestingly, increase in fiber diameter from 335 ± 154 nm to 3013 ± 531 nm resulted in enhanced myoblast proliferation and differentiation as well as myoblast fusion into mature myotubes indicating the ability of cells to respond to fiber topography. *In vivo* study using an *mdx* mouse model demonstrated the promise of using optimized fiber scaffolds to improve myogenic potential of transplanted cells. Taken together, our studies demonstrated that biomimetic fiber scaffolds through topographical contact guidance enhanced myogenic potential of myoblasts; hence, providing critical insights into harnessing scaffold physical properties for accelerated muscle healing.

## Data Availability Statement

All datasets generated for this study are included in the article/Supplementary Material.

## Ethics Statement

The animal study was reviewed and approved by Purdue University's Animal Care and Use Committee.

## Author Contributions

NN and MD conceived and designed the experiments, and prepared the manuscript. NN, CJ, and NW performed the *in vitro* cell culture experiments. DC and OJ contributed to the nanomechanical characterization of the fibers. NN, CW, GU, and SK contributed to the *in vivo* experimental design and analysis. All authors discussed the results and commented on the manuscript.

### Conflict of Interest

The authors declare that the research was conducted in the absence of any commercial or financial relationships that could be construed as a potential conflict of interest.
