# Supplementary material for: Harnessing Fiber Diameter-Dependent Effects of Myoblasts Toward Biomimetic Scaffold-Based Skeletal Muscle Regeneration
Source: Front Bioeng Biotechnol. 2020 Mar 24;8:203. doi: 10.3389/fbioe.2020.00203 (PMC7105569; doi:10.3389/fbioe.2020.00203)
Supplement: Supplementary file 1 [file Table_1.docx]

Supplementary Material

# Supplementary Data

*Custom-Designed Electrospinning Set-up*

To improve the alignment of the deposited fibers, we custom-designed a novel electrospinning collector with digitated metal blades. These equally spaced blades provide a uniform local electric field between them, to facilitate the alignment of the deposited fibers. Fiber deposited within the blades were characterized and used for the study.

*Nanoindentation Characterization*

Electrospun fibers were collected on a clean cover slip (spun for 10 min to obtain sparsely distributed single fibers with limited overlapping). Deionized water (2 mL) was added onto the sample, removed with filter paper, and air dried to tightly fix the fibrils to the cover slip. The fibers were then imaged with the Asylum MFP-3D atomic force microscope (AFM) operating under AC mode with a scan rate of 1 Hz, equipped with a TAP-AI 150 probe (Budget Sensor). Nanoindentation measurements of the fibers were performed with the same probe. The spring constant of the probe was pre-calibrated by the thermal tune method before indenting. The loading and unloading velocity were kept at 100 nm/s throughout the measurement and the deflection of the cantilever was set at 200 nm, resulting in an indentation depth of 30-50 nm. At least 15 force-distance curves were collected for each fiber at different locations and a minimum of 5 different fibers were examined for each sample. Young’s modulus of the electrospun fibers was obtained by fitting the unloading curve of force-vs-indentation depth graph using Oliver-Pharr model, with the ε=0.75 and β= 1.034 [1] for tip geometry (Berkovich shape), and Poisson ratio of 0.33 (assumed value) for PLGA. Only first 75% of the unloading curve was considered in fitting to better reveal the surface mechanical properties of the electrospun fibers.

*FAK Analysis for Myoblast Adhesion*

To understand the underlying cell adhesion mechanism, we cultured cells on aligned PLGA fibers, random PLGA fibers and tissue culture plastics (TCPS), and evaluated for their pFAK levels at 2 h post seeding. Collected samples were rinsed with phosphate buffer saline and then minced in RIPA buffer to extract the proteins. After centrifugation, supernatant was collected and processed for western blot analysis (fiber debris accumulated in the pellet). Samples were analyzed for Phospho-FAK (Tyr397) (Cell signaling technology; 1:1000 dilution) and GAPDH (Santa Cruz Biotechnology; 1:100 dilution).

*SEM of Myoblasts on Fibers*

To assess the myoblast growth on the fabricated aligned PLGA fibers, C2C12 cells were seeded on the fibers and allowed to proliferate for seven days. At predetermined time points, samples were collected, rinsed with phosphate buffer saline, fixed in 4% and dehydrated in series of ethanol before processing the sample for SEM analysis.

[1] A.-Y. Jee, M. Lee, *Polymer Testing* **2010**, *29*, 95-99.

# Supplementary Figures

Figure S1


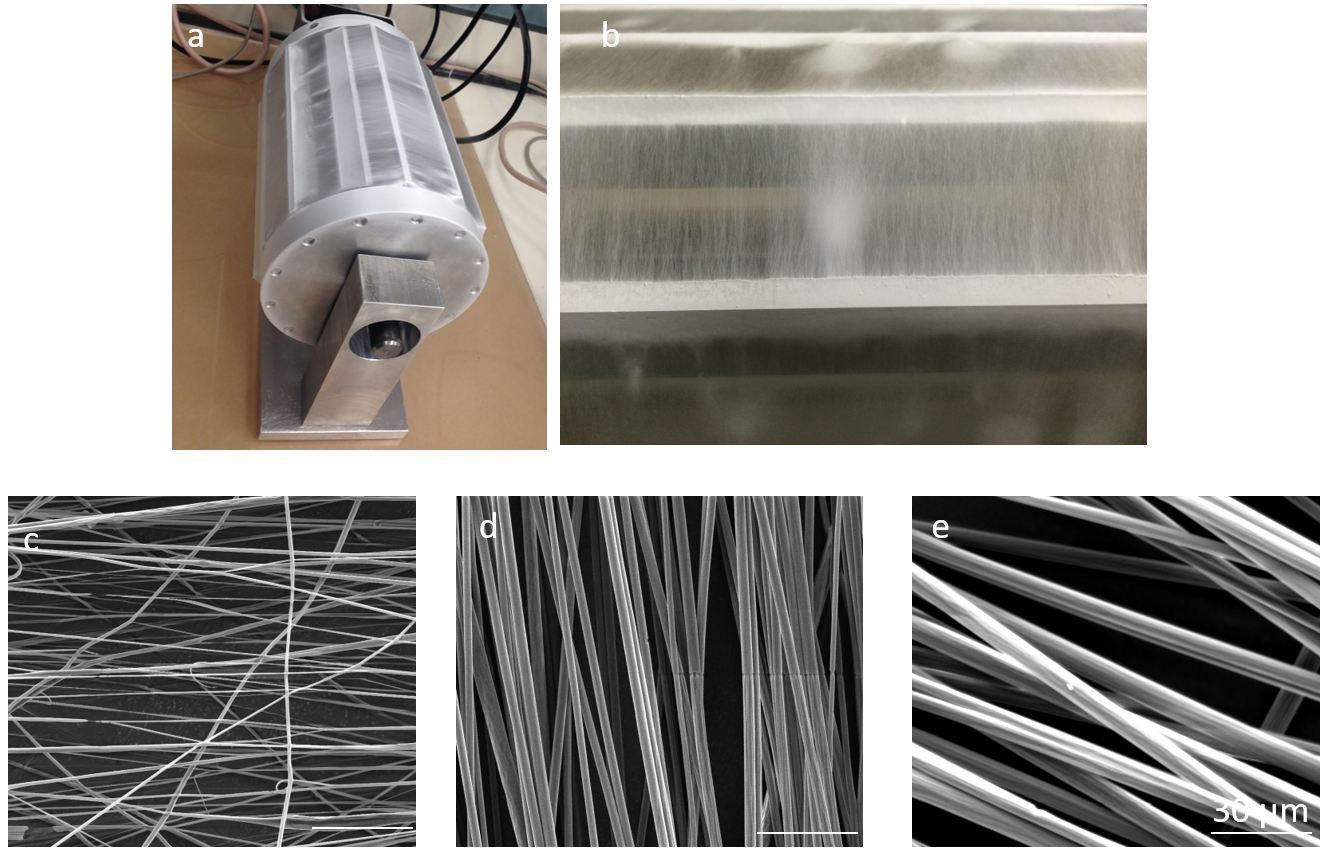


**Figure S1**: a) Custom-designed rotating collector of electrospinning with b) aligned fibers collected between the interspaced parallel blades. SEM images of aligned fibers with different polymer concentrations c) 20% PLGA (Matrix 1); (d) 30% PLGA (Matrix 2); (e) 40% fibers (Matrix 3).

Figure S2

**
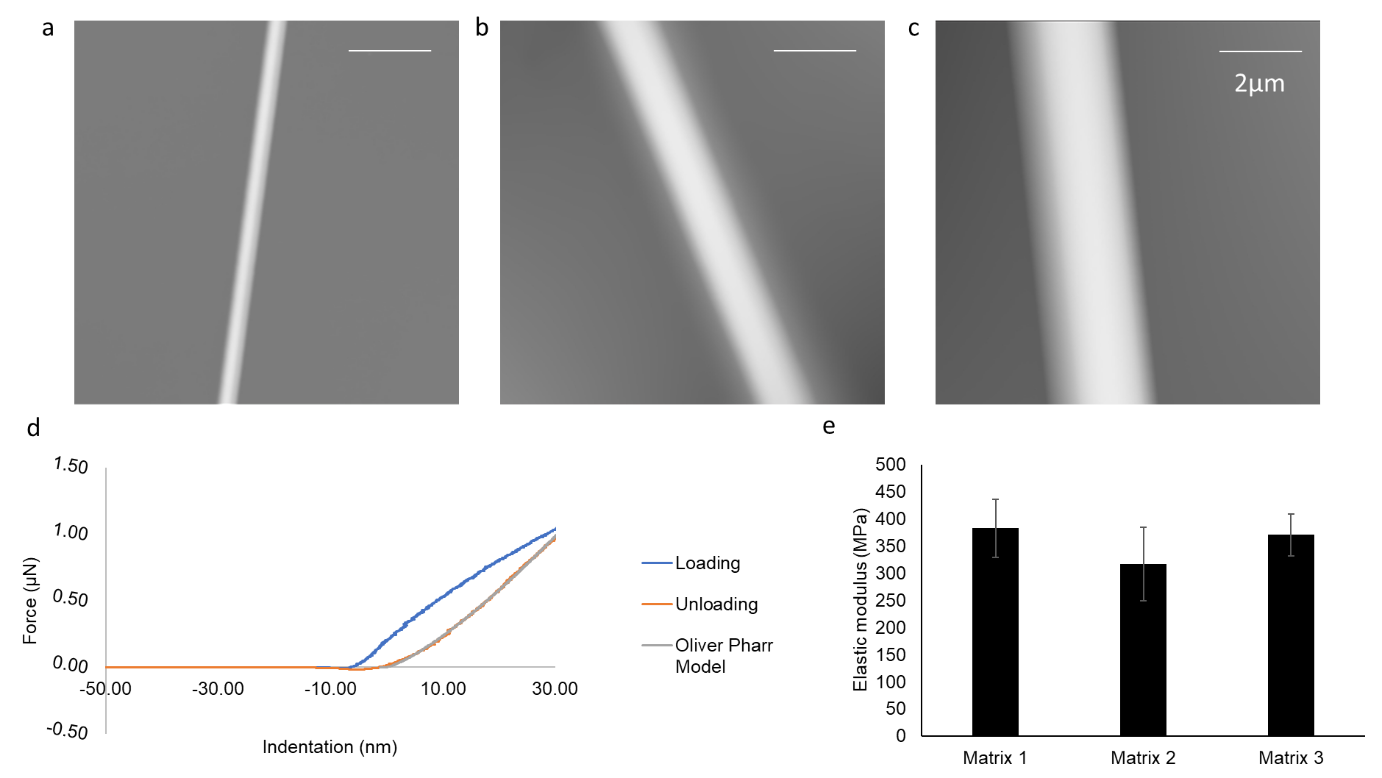
**

**Figure S2.** AFM topography images of single fibers observed in a) Matrix 1, b) Matrix 2 and c) Matrix 3. d) Representative loading, unloading and model fit plot for the nanoindentation force-distance curve on an electrospun single fiber. e) Bar graph representing the calculated elastic modulus based on Oliver Pharr model for Matrix 1, Matrix 2 and Matrix 3.

Figure S3


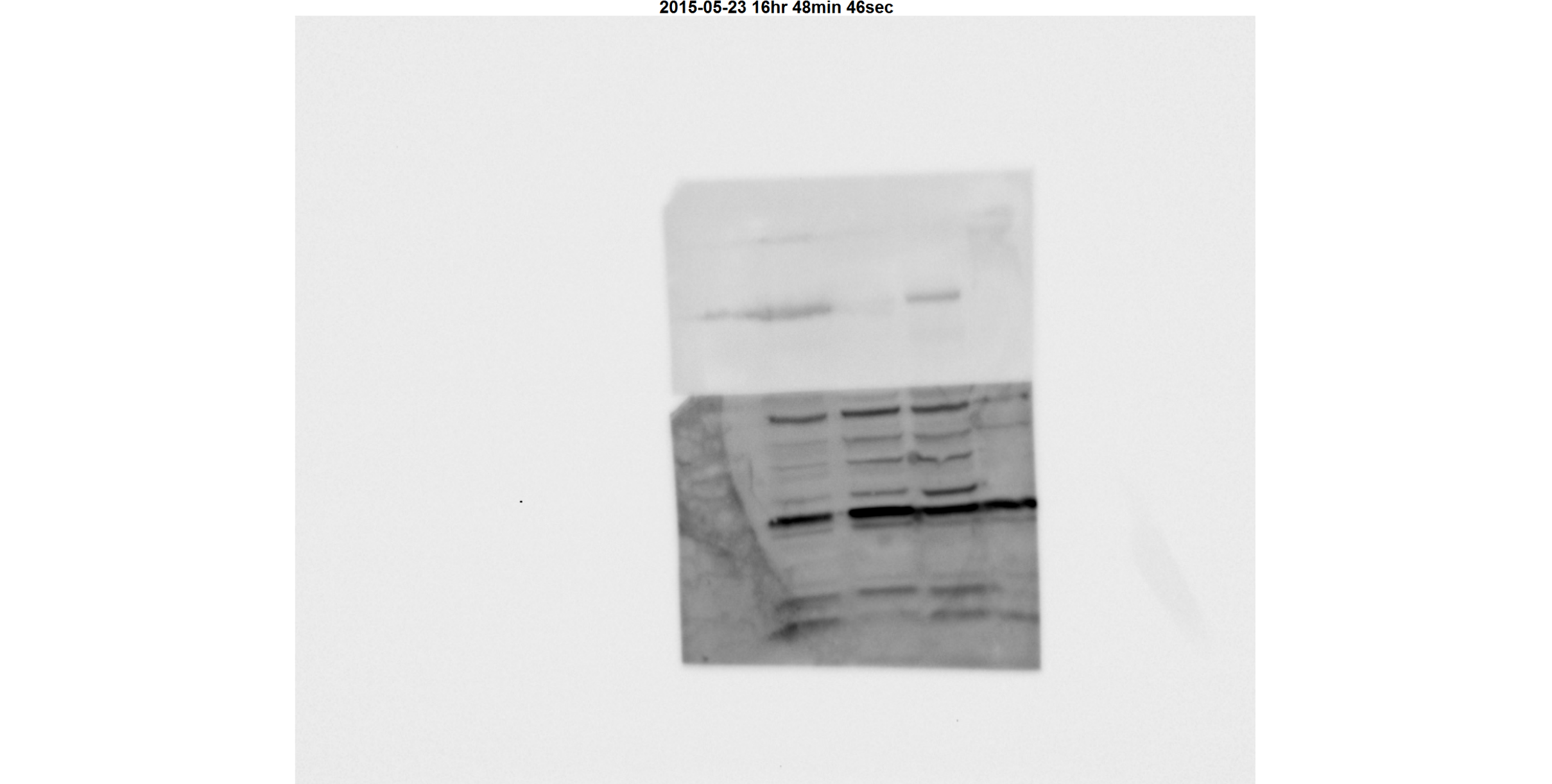


GAPDH

pFAK

control

Matrix 2

random

**Figure S3**: Western blot analysis for pFAK indicating elevated expression levels for aligned fibers (Matrix 2) compared to random fibers.

Figure S4


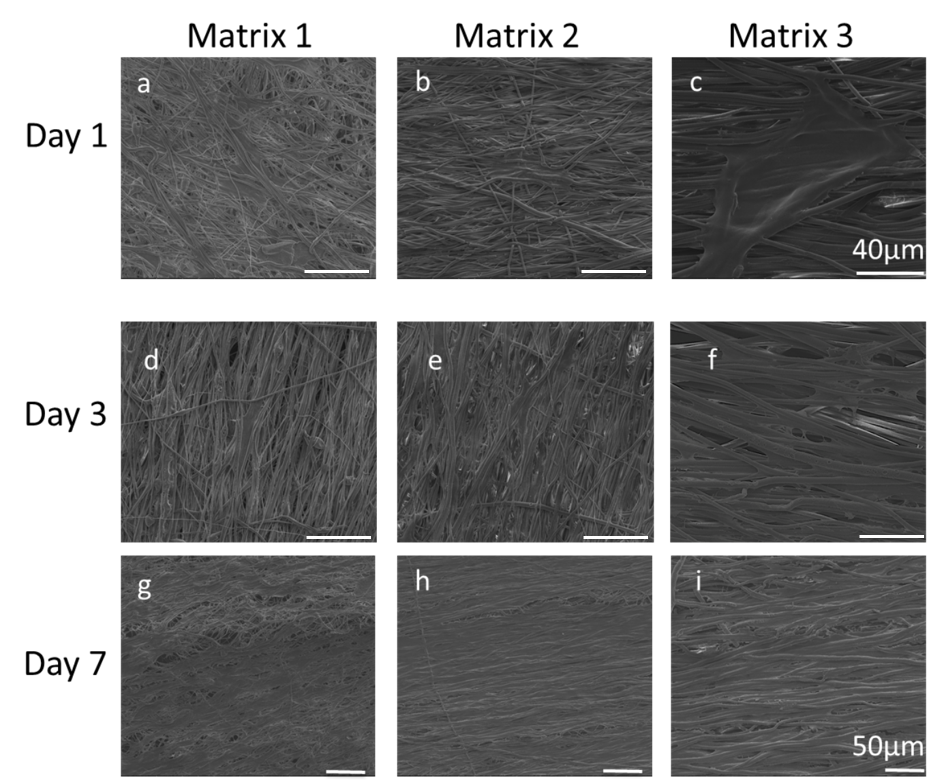


**Figure S4**: Representative SEM micrographs of myoblasts at day 1 (top row) on a) Matrix 1, b) Matrix 2, and c) Matrix 3; at day 3 (middle row) on d) Matrix 1, e) Matrix 2 and f) Matrix 3; and at day 7 (bottom row) on g) Matrix 1, h) Matrix 2, i) Matrix 3. Scale bar: 40 µm for images a-f, Scale bar: 50 µm for images g-i.

Figure S5


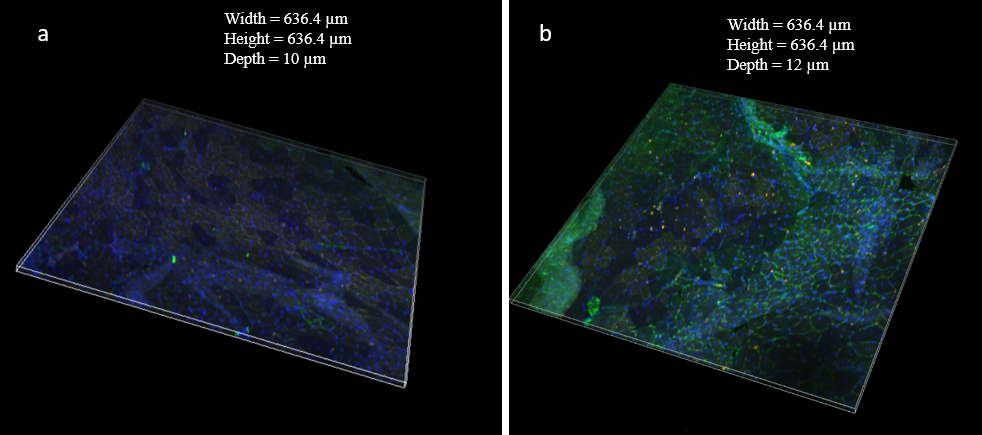


Figure S5: Z-stack images of immunostaining for dystrophin for the TA muscles 21 days post implantation for both a) myoblast transplantation alone (controls) and b) fiber scaffolds seeded with primary myoblasts.
